# Supplementary material for: Inter-Observer Agreement in Radiographic Diagnosis of Coxofemoral Joint Disease in a Closed Cohort of Four-Month-Old Rottweilers
Source: Animals (Basel). 2022 May 15;12(10):1269. doi: 10.3390/ani12101269 (PMC9137964; doi:10.3390/ani12101269)
Supplement: Supplementary file 1 [file animals-12-01269-s001.zip › animals-1692126-supplementary.pdf]

Supplementary Materials:

Figure S1: The results of the general linear model and Bonferroni's alpha correction procedure of the quantitative data.

Multiple Comparisons

Bonferroni

Dependent Variable	(I) Examiner	(J) Examiner	Mean Difference (I-J)	Std. Error	Sig.	95% Confidence Interval Lower Bound	95% Confidence Interval Upper Bound
NA	1	2	2.5395	1.33631	.585	-1.2437	6.3227
		3	3.6352	1.34840	.075	-.1823	7.4526
		4	.1310	1.35474	1.000	-3.7044	3.9664
		5	-.0669	1.38237	1.000	-3.9805	3.8467
	2	1	-2.5395	1.33631	.585	-6.3227	1.2437
		3	1.0957	1.33631	1.000	-2.6875	4.8789
		4	-2.4085	1.34271	.740	-6.2098	1.3929
		5	-2.6064	1.37058	.583	-6.4866	1.2739
	3	1	-3.6352	1.34840	.075	-7.4526	.1823
		2	-1.0957	1.33631	1.000	-4.8789	2.6875
		4	-3.5042	1.35474	.102	-7.3396	.3313
		5	-3.7021	1.38237	.079	-7.6157	.2115
	4	1	-.1310	1.35474	1.000	-3.9664	3.7044
		2	2.4085	1.34271	.740	-1.3929	6.2098
		3	3.5042	1.35474	.102	-.3313	7.3396
		5	-.1979	1.38856	1.000	-4.1291	3.7332
	5	1	.0669	1.38237	1.000	-3.8467	3.9805
		2	2.6064	1.37058	.583	-1.2739	6.4866
		3	3.7021	1.38237	.079	-.2115	7.6157
		4	.1979	1.38856	1.000	-3.7332	4.1291
DI	1	2	.0204	.03031	1.000	-.0654	.1062
		3	-.0267	.03058	1.000	-.1132	.0599
		4	-.0007	.03073	1.000	-.0877	.0863
		5	-.0122	.03135	1.000	-.1010	.0765
	2	1	-.0204	.03031	1.000	-.1062	.0654
		3	-.0471	.03031	1.000	-.1329	.0387
		4	-.0211	.03045	1.000	-.1074	.0651
		5	-.0327	.03109	1.000	-.1207	.0554
	3	1	.0267	.03058	1.000	-.0599	.1132
		2	.0471	.03031	1.000	-.0387	.1329
		4	.0260	.03073	1.000	-.0610	.1129
		5	.0144	.03135	1.000	-.0743	.1032

	4	1	.0007	.03073	1.000	-.0863	.0877
		2	.0211	.03045	1.000	-.0651	.1074
		3	-.0260	.03073	1.000	-.1129	.0610
		5	-.0115	.03149	1.000	-.1007	.0776
	5	1	.0122	.03135	1.000	-.0765	.1010
		2	.0327	.03109	1.000	-.0554	.1207
		3	-.0144	.03135	1.000	-.1032	.0743
		4	.0115	.03149	1.000	-.0776	.1007
DARS	1	2	-.8742	.88747	1.000	-3.3867	1.6383
		3	-4.2574*	.89550	.000	-6.7926	-1.7222
		4	-.3497	.89971	1.000	-2.8969	2.1975
		5	2.8741*	.91806	.019	.2750	5.4733
	2	1	.8742	.88747	1.000	-1.6383	3.3867
		3	-3.3832*	.88747	.002	-5.8957	-.8707
		4	.5245	.89172	1.000	-2.0001	3.0490
		5	3.7483*	.91022	.001	1.1714	6.3253
	3	1	4.2574*	.89550	.000	1.7222	6.7926
		2	3.3832*	.88747	.002	.8707	5.8957
		4	3.9077*	.89971	.000	1.3606	6.4549
		5	7.1316*	.91806	.000	4.5324	9.7307
	4	1	.3497	.89971	1.000	-2.1975	2.8969
		2	-.5245	.89172	1.000	-3.0490	2.0001
		3	-3.9077*	.89971	.000	-6.4549	-1.3606
		5	3.2238*	.92217	.006	.6131	5.8346
	5	1	-2.8741*	.91806	.019	-5.4733	-.2750
		2	-3.7483*	.91022	.001	-6.3253	-1.1714
		3	-7.1316*	.91806	.000	-9.7307	-4.5324
		4	-3.2238*	.92217	.006	-5.8346	-.6131
CEA	1	2	-.8933	1.01812	1.000	-3.7756	1.9891
		3	.9537	1.02733	1.000	-1.9548	3.8622
		4	2.0379	1.03217	.494	-.8842	4.9601
		5	.7991	1.05321	1.000	-2.1827	3.7808
	2	1	.8933	1.01812	1.000	-1.9891	3.7756
		3	1.8470	1.01812	.708	-1.0354	4.7293
		4	2.9312*	1.02300	.045	.0350	5.8274
		5	1.6923	1.04423	1.000	-1.2640	4.6487
	3	1	-.9537	1.02733	1.000	-3.8622	1.9548
		2	-1.8470	1.01812	.708	-4.7293	1.0354
		4	1.0842	1.03217	1.000	-1.8379	4.0064
		5	-.1546	1.05321	1.000	-3.1364	2.8271
	4	1	-2.0379	1.03217	.494	-4.9601	.8842
		2	-2.9312*	1.02300	.045	-5.8274	-.0350

	3	-1.0842	1.03217	1.000	-4.0064	1.8379
	5	-1.2389	1.05793	1.000	-4.2340	1.7562
5	1	-.7991	1.05321	1.000	-3.7808	2.1827
	2	-1.6923	1.04423	1.000	-4.6487	1.2640
	3	.1546	1.05321	1.000	-2.8271	3.1364
	4	1.2389	1.05793	1.000	-1.7562	4.2340

Based on observed means.

The error term is Mean Square(Error) = 28.496.

*. The mean difference is significant at the .05 level.

Figure S2: The results of Cohen's kappa coefficient for qualitative values. The values with a kappa below 0.20 indicate poor agreement. The values between 0.21 and 0.40 indicate weak agreement. The kappa between 0.41 and 0.60 indicates moderate and 0.61 to 0.80 indicates good agreements. The results with a kappa above 0.80 indicate excellent agreement.

**SCAR**	Observer 1	Observer 2	Observer 3	Observer 4	Observer 5
Observer 1		0	0	0	0
Observer 2	0		0.14	0.07	0.35
Observer 3	0	0.14		0.06	0.03
Observer 4	0	0.07	0.06		0.41
Observer 5	0	0.35	0.03	0.41	

**LCFH**	Observer 1	Observer 2	Observer 3	Observer 4	Observer 5
Observer 1		0.57	0.40	0.49	0.50
Observer 2	0.57		0.36	0.46	0.46
Observer 3	0.40	0.36		0.27	0.25
Observer 4	0.49	0.46	0.27		0.54
Observer 5	0.50	0.46	0.25	0.54	

**GDJD**	Observer 1	Observer 2	Observer 3	Observer 4	Observer 5
Observer 1		0.11	0.12	0.14	0.11
Observer 2	0.11		0	0.29	0.04
Observer 3	0.12	0		0.15	0.18
Observer 4	0.14	0.29	0.15		0.03
Observer 5	0.11	0.04	0.18	0.03	

**GDAR**	Observer 1	Observer 2	Observer 3	Observer 4	Observer 5
Observer 1		0.09	0.04	0.09	0.02
Observer 2	0.09		0.02	0.19	0.03
Observer 3	0.04	0.02		0.03	0.10
Observer 4	0.09	0.19	0.03		0.21
Observer 5	0.02	0.03	0.10	0.21	
